# A narrative review of house dust mite allergy: species distribution and allergen sensitisation patterns across tropical regions

**DOI:** 10.3389/falgy.2026.1813656

**Published:** 2026-05-21

**Authors:** Hiryahafira Mohamad Tahir, Nik Abdul Aziz Nik Kamarudin, Mariana Ahamad, Ernieenor Faraliana Che Lah

**Affiliations:** Acarology Unit, Infectious Diseases Research Centre, Institute for Medical Research (IMR), National Institutes of Health, Ministry of Health Malaysia, Shah Alam, Malaysia

**Keywords:** allergen sensitisation, allergy diagnostics, house dust mite allergy, skin prick test, tropical regions

## Abstract

House dust mite (HDM) allergy is among the leading causes of allergic disease worldwide, yet significant knowledge gaps regarding its ecology, prevalence, and diagnostic approaches in tropical regions remain unexplored. The constant warmth and humidity characteristic of the tropics create optimal conditions for mite proliferation and year-round allergen exposure, leading to high sensitisation rates among populations with allergies. This review synthesises current knowledge on the ecology, distribution, and sensitisation patterns of HDM species in tropical environments, with particular attention to *Dermatophagoides pteronyssinus*, *Dermatophagoides farinae*, and *Blomia tropicalis*. It also examines diagnostic approaches used across tropical countries, ranging from conventional skin prick testing (SPT), specific IgE test and to advanced molecular methods such as Component-Resolved Diagnosis (CRD). By integrating ecological and clinical perspectives, this review highlights the importance of region-specific research and diagnostic strategies that account for species diversity and cross-reactivity unique to tropical settings. Advancing molecular allergen characterisation and developing standardised tropical mite extracts will be essential for improving diagnostic accuracy, guiding immunotherapy, and informing public health policies aimed at reducing the burden of mite-related allergic diseases in tropical populations.

## 1 Introduction

Allergic diseases are chronic, immune-mediated disorders resulting from hypersensitivity reactions to environmental allergens (1). Allergy-related symptoms include food allergy, asthma, atopic dermatitis (AD), allergic rhinitis (AR), conjunctivitis, angioedema, urticaria, eczema, eosinophilic disorders and allergies to drugs and insects (2, 3). The global prevalence of allergic diseases continues to rise, with an estimated 300 million people affected by asthma, 200–250 million by food allergies, 400 million by AR, and approximately 10% of the population experiencing drug allergies (4). These conditions often co-exist within the same individual and show no signs of declining prevalence (2, 5). This growing burden highlights the need for a comprehensive diagnostic and therapeutic approach, alongside greater awareness among healthcare providers and the general public (2).

Most allergic diseases are initiated or exacerbated by aeroallergens, airborne substances that can induce hypersensitivity reactions in susceptible individuals. These include pollens, fungal spores and house dust mites (HDMs) (6–8). Among these, HDMs have emerged as one of the most clinically significant sources of allergens worldwide, particularly in respiratory allergies such as asthma and AR (9). Dust mites are microscopic arachnids belonging to the order Acari that are ubiquitous in human dwellings and present in nearly all inhabited regions of the world (10). Based on their ecological niches, mites are broadly classified into house dust mites (HDMs) and storage mites (SMs). HDMs predominantly inhabit indoor environments such as bedding, mattresses, carpets, and upholstered furniture, whereas SMs are commonly associated with stored food products, grains, and other organic materials (11, 12). Both groups are clinically relevant allergen sources capable of inducing IgE-mediated sensitisation but SMs remain poorly characterised in terms of their allergenic potential and clinical relevance (10). It is estimated that clinically confirmed HDM allergy affects approximately 4% and 6% of the global population, representing approximately 500 million individuals (13), and sensitisation to HDM allergens is strongly linked to the development and persistence of allergic respiratory diseases, underscoring their significant clinical impact (2).

Globally, *Dermatophagoides pteronyssinus* and *Dermatophagoides farinae*, members of the family Pyroglyphidae are considered the predominant HDM species and are frequently included in diagnostic and therapeutic preparations (14). However, in tropical and subtropical regions, *Blomia tropicalis* (Family: Glycyphagidae) frequently emerges as a dominant species and, in some populations, surpasses *Dermatophagoides* in sensitisation rates (15–17). These observations suggest that the distribution of mite species and their clinical relevance are influenced by geography and climate. This geographic variation is further supported by a comparative study that analysed IgE sensitisation profiles to 17 HDM components in allergic adults across Canada, Europe, South Africa, and the United States (18). The study highlighted significant regional variability in HDM sensitisation profiles, indicating that the prevalence of IgE reactivity to specific HDM allergens is influenced by geographic factors. In addition, a large population-based study in Türkiye using molecular IgE profiling with microarrayed allergens demonstrated distinct interregional sensitisation patterns across different geographic locations (19). The findings suggested that variations in environmental exposure and regional characteristics may influence allergen sensitisation profiles. Interestingly, one region (Kayseri) was identified as a hypoallergenic area, with significantly lower levels of allergen sensitisation compared with other regions.

**Figure 1:**
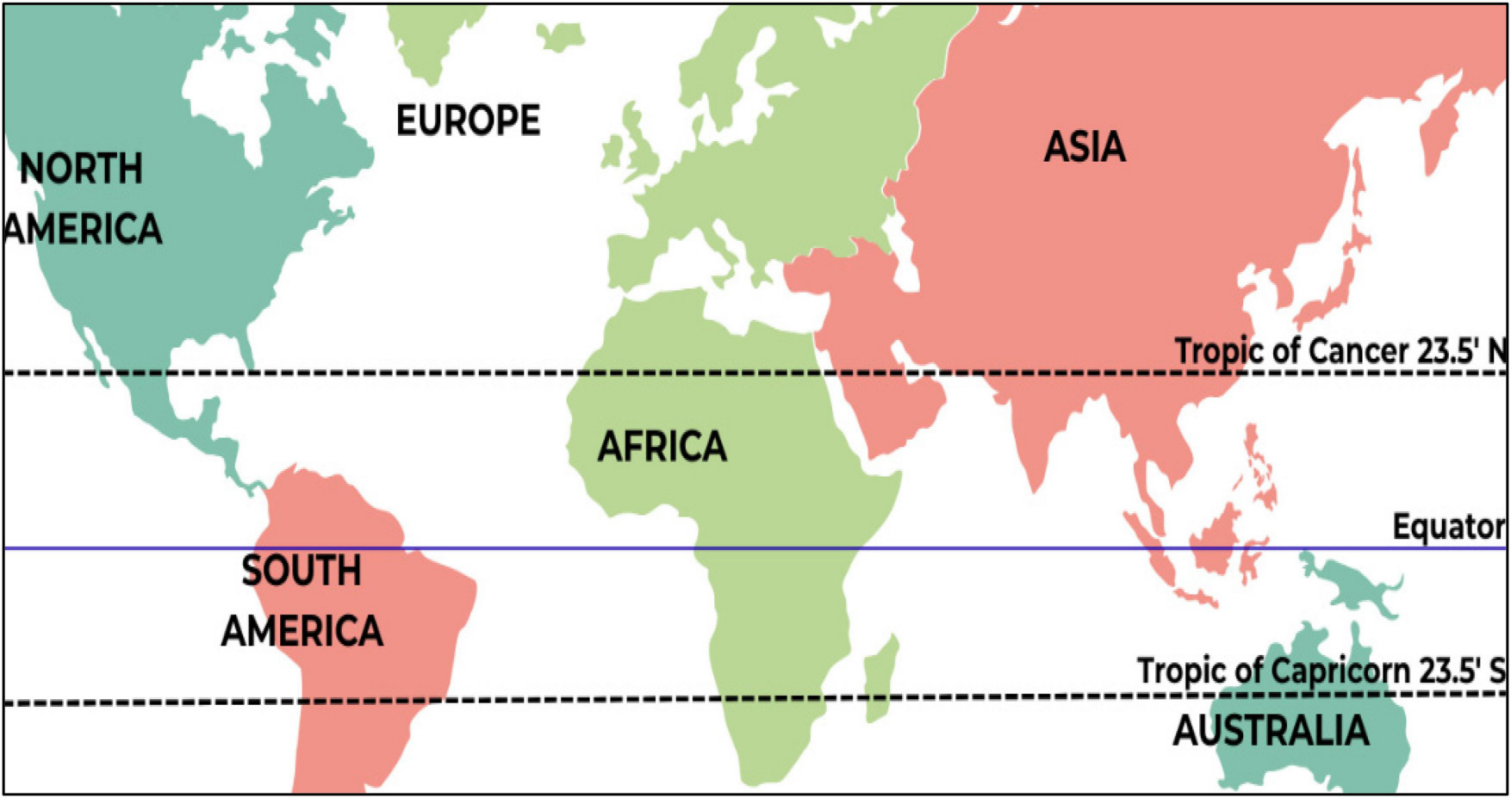
Global map highlighting the tropical region between the tropic of cancer (23.5°N) and the Tropic of Capricorn (23.5°S). This region encompasses the primary geographic focus of this review on house dust mite allergy.

The warm and humid climate of tropical regions provides ideal conditions for HDM growth and reproduction, leading to year-round exposure and a higher risk of sensitisation. Figure 1 shows that the tropical regions lie between the Tropic of Cancer (23.5° N) and the Tropic of Capricorn (23.5° S), characterised by a mean annual temperature of approximately 28 °C and relative humidity around 85% (20). Based on these climatic and ecological characteristics, the tropical belt encompasses countries in Central and South America, Africa, South Asia, Southeast Asia, the tropical edges of East Asia, and northern Australia, as well as the Pacific Islands.

Allergy research in tropical regions reveals unique clinical and immunological features driven by environmental and climatic factors, warranting special attention from both the scientific community and clinicians, particularly in relation to HDM allergy (20). Accurate diagnosis of HDM allergy relies on objective testing, as clinical symptoms alone are non-specific and cannot reliably distinguish allergic from non-allergic conditions (21). While Skin prick testing (SPT) remains the most widely used and practical method in tropical regions, the incorporation of *in vitro* assays and molecular approaches such as ImmunoCAP and CRD is essential for identifying species-specific sensitisation and improving diagnostic precision in areas with diverse mite exposures (22, 23).

Despite the high potential for HDM proliferation, studies on house dust mite allergy in tropical countries remain comparatively limited and fragmented, often confined to individual nations rather than providing a regional perspective. Consequently, important questions remain regarding species prevalence, allergen diversity, co-sensitisation patterns, and their implications for accurate diagnosis and effective treatment (5, 24). In addition to HDMs, SMs such as *Tyrophagus putrescentiae*, *Acarus siro*, and *Suidasia* spp. are commonly found in tropical household dust and stored products (15, 25–27). Yet these species are rarely included in standard diagnostic panels, potentially leading to an underestimation of their contribution to allergic disease in tropical populations. To date, no comprehensive analysis has systematically summarised the distribution of HDMs species, allergen sensitisation patterns, and diagnostic approaches for HDM allergy across tropical regions.

Therefore, this review summarises current knowledge on the distribution and sensitisation of house dust mite allergy in tropical regions, including species ecology, prevalence patterns and diagnostic approaches. By integrating and highlighting current research on tropical HDM allergy, this review provides a comprehensive understanding intended to guide future studies to improve diagnostic accuracy, inform immunotherapy development, and shape public health interventions.

## 2 Methodology of literature review

The narrative review methodology was chosen because it allows for a comprehensive synthesis of diverse study types ranging from environmental entomology to clinical immunology, providing a broader conceptual framework for understanding mite distribution and its clinical impact than a strict systematic review would permit. To ensure a transparent and structured approach, a literature search was conducted across three primary electronic databases: PubMed, Google Scholar, and Scopus. The search strategy employed combinations of Boolean terms and keywords, including “dust mite”, “house dust mites”, 'storage mite', “house dust mite allergy”, “IgE sensitisation”, “allergen sensitisation”, “allergy diagnostics”, or 'skin prick test'.

To maintain high scientific rigor, the inclusion criteria were limited to articles published in the English language between 1990 and 2026. Furthermore, a geographic filter was applied to prioritize studies conducted in tropical regions to account for regional variations in mite prevalence and sensitisation profiles. The selection process involved rigorous manual screening of titles and abstracts to ensure that the findings contributed specifically to understanding house dust mite (HDM) prevalence and clinical sensitisation in tropical environments. Articles that did not provide clear species identification or fell outside the defined geographical scope were excluded.

## 3 Ecology and distribution of house dust mite species in the tropics

**Figure 2:**
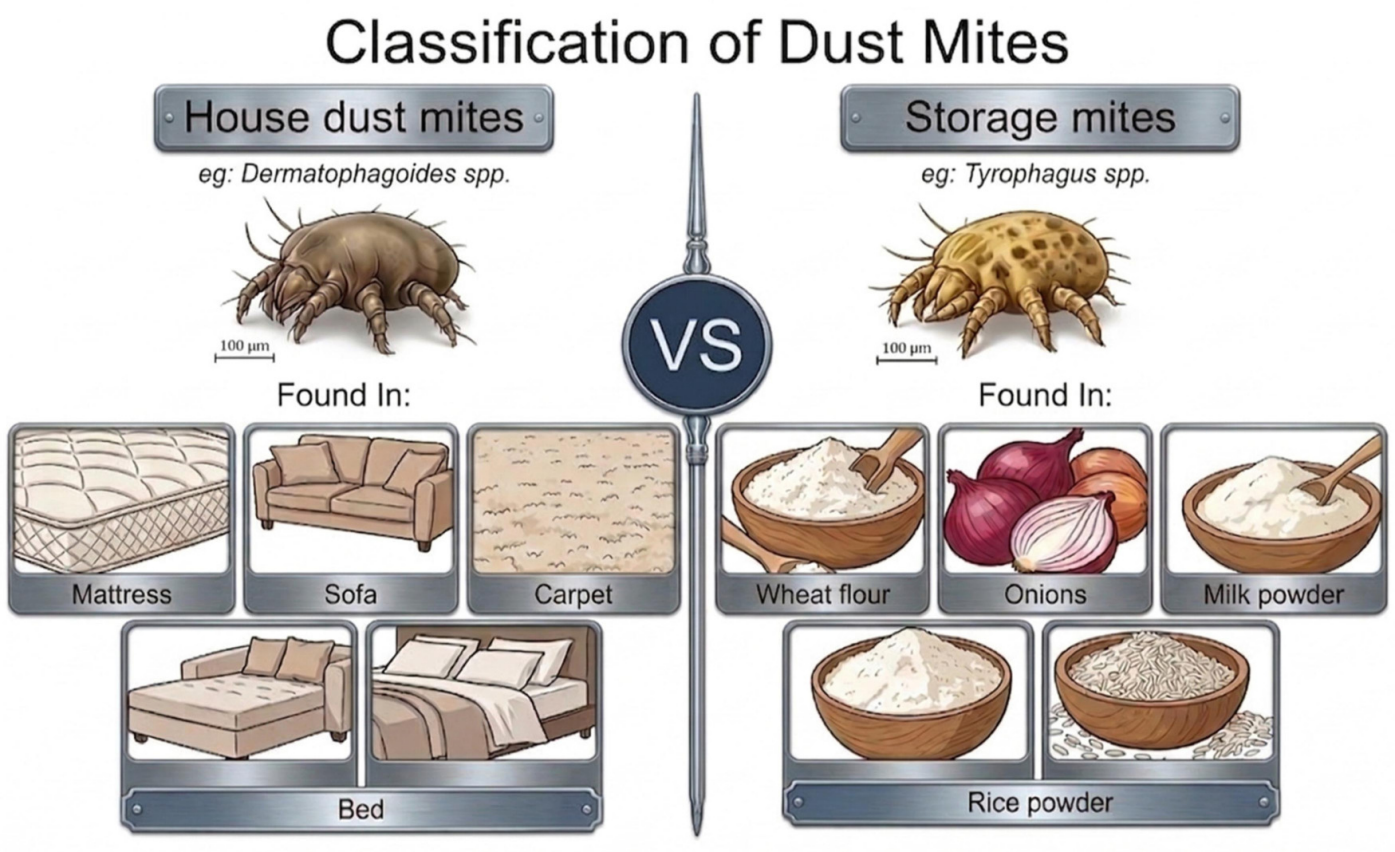
Classification of house dust mites and storage mites based on their common habitats and sources.

Dust mites are cosmopolitan arthropods that have successfully adapted to diverse indoor and storage environments worldwide (28). Figure 2 shows the classification of dust mite that HDMs predominantly occupy domestic indoor microhabitats, including bedding, mattresses, carpets, and upholstered furnishings, whereas storage mites (SMs) are primarily associated with stored grains, food products, and other organic substrates (12, 13). Their distribution and abundance are strongly influenced by ecological factors such as temperature (optimal temperature at 25–30 °C), 75%–80% relative humidity, and availability of organic substrates, which provide both food and microhabitats (10). The persistently warm, humid climate across tropical regions sustains mite populations, resulting in continuous allergen presence throughout the year (20). Studies on the ecology and geographic distribution of dust mites are essential for understanding patterns of allergen exposure, as mite abundance, species composition, and habitat preferences directly influence the types and intensities of allergens encountered by human populations.

In tropical regions, the geographical distribution of dust mites is dominated by species from the family Pyroglyphidae, comprising *D. pteronyssinus* and *D. farinae*, as well as species from the family Glycyphagidae, such as *B. tropicalis.* Table 1 summarises the reported diversity and distribution of dust mites that are present in the tropical region detected using microscopy or enzyme-linked immunosorbent assays (ELISA).

**Table 1:** Diversity and distribution of dust mites in the tropical region.

Tropical region	Country	Sampling sites	Temperature/Relative Humidity	Species HDM/Allergen Types	Method detection	References
Southeast Asia	Malaysia	Mattresses in 20 houses in the Klang Valley	Temperature: Not mentioned Humidity: Not mentioned	➢ *Blomia tropicalis*^a^ ➢ *Austroglycyphagus malaysiensis* ➢ *Trophilicus aframericanus* ➢ *Acarus* spp. ➢ *Tyrophagus putrescentiae* ➢ *Chortoglyphus arcuatus* ➢ *D. farinae* ➢ *D. pteronyssinus* ➢ *Euroglyphus maynei* ➢ *Hirstia domicola* ➢ *Malayoglyphus intermedius* ➢ *Sturnophagoides brassiliensis* ➢ *Suidasia nesbitti* ➢ *S. pontifica* ➢ *Suidasia* spp.	• Microscopy	(11)
		Edible bird nest products	Temperature: Not mentioned Humidity: Not mentioned	➢ *Suidasia sp.* ➢ *Austroglycyphagus sp*. ➢ *Aleuroglyphus ovatus*	• Microscopy	(29)
		Rice flour	Temperature: Not mentioned Humidity: Not mentioned	➢ *S. pontifica* ➢ *T. putrescentiae*	• Microscopy	(12)
		Classroom of junior high schools in Johor Bahru	Temperature: 29 °C. Humidity: 70%	Low level of dust mite allergens (77 ng/g dust) ➢ Der p 1 ➢ Der f 1 ➢ Der m 1 ➢ Blo t	• ELISA	(30)
		Milk powder	Temperature: Not mentioned Humidity: Not mentioned	➢ *S. pontifica*	• Microscopy	(25)
		Administrative Office of Universiti Putra Malaysia	Temperature: Not mentioned Humidity: Not mentioned	➢ Der p 1 (556 ng/g dust) ➢ Der f 1 (658 ng/g dust)	• ELISA	(31)
	Philippines	Living room floors of houses		➢ *B. tropicalis*^a^ ➢ *C. arcuatus* ➢ *Austroglycyphagus sp.* ➢ *Lepidoglyphus destructor* ➢ *Glycyphagus domesticus* ➢ *G. privatus* ➢ *D. farinae*^a^ ➢ *D. pteronyssinus* ➢ *E. maynei* ➢ *S. brasiliensis* ➢ *M. intermedius* ➢ *M. carmelitus* ➢ *Acarus sp.* ➢ *Tyrophagus sp.* ➢ Der 2 (low levels of antigens detected)	• Microscopy • ELISA (only species *Dermatophagoides*)	(32)
	Thailand	Mattresses of the House officers’ dormitory at Siriraj hospital	Mean Temperature: 29 °C. Mean Humidity: 60.6%	➢ Der p 1 ➢ Der f 1^a^	• ELISA	(33)
		Cooking flour	Temperature: Not mentioned Humidity: Not mentioned	➢ *D. farinae*	➢ Microscopy	(34)
		Mattress and living room floor	Temperature: Not mentioned Mean Humidity: 42% -62%	➢ Der p 1 ➢ Der f 1	➢ ELISA	(35)
	Singapore	Floor, mattress, sofa, pillows, carpets and curtains in hospital	Temperature: Not mentioned Humidity: Not mentioned	➢ Der p 1^a^ ➢ Der f 1	➢ ELISA	(36)
		Wheat Flour	Temperature: 26 °C. Humidity: Not mentioned	➢ *D. farinae*	➢ Microscopy	(37)
		Floors in child care centres	Temperature: 21–31 °C. Humidity: 60–80%	➢ Der p 1 ➢ Blo t 5	• BioPlex 2200 (Der P 1) • ELISA (Blo t 5)	(38)
		Houses (mattresses, sofas, carpets and bedroom floors)	Temperature: Not mentioned Humidity: Not mentioned	➢ *Blomia tropicalis*^a^ ➢ *D. pteronyssinus* ➢ *Sturnophagoides brassiliensis* ➢ *T. granarius* ➢ *D. farinae* ➢ *A. malaysiensis* ➢ *C. malaccensis* ➢ *M. intermedius* ➢ *S. pontifica* ➢ *T. putrescentiae* ➢ *T. granarius*	➢ Microscopy	(15)
South Asia	South Assam, India	Houses (floor and bed dust) of atopic patients	Temperature: 29–32 °C. Humidity: 90–94%	➢ *Dermatophagoides* spp.^a^ ➢ *Blomia* spp. ➢ *Acarus* spp. ➢ *Cheyletus* spp. ➢ *Campylochirus* spp. ➢ *Caloglyphus* spp.	➢ Microscopy	(26)
Southwest China	Xishuangbanna Dai, Southwest China	Houses of allergic patients (pillows, quilts, sheets, sleeping pads and mattresses, sofas, rugs, and carpet floors)	Temperature: Not mentioned Humidity: Not mentioned	➢ *D. farinae*^a^ ➢ *D. pteronyssinus* ➢ *D. siboney* ➢ *T. putrescentiae* ➢ *A. ovatus* ➢ *B. tropicalis*	• Microscopy	(27)
South America	Columbia	Houses of allergic patients (mattresses and the floor)	Temperature: 27.0 °C to 28 °C Humidity: 77% to 82%.	➢ *B. tropicalis*	➢ Radioallergosorbent test (RAST) inhibition	(39)
	Ecuador	Houses (mattresses and carpet)	Mean Temperature: 16.0 °C to 24 °C Mean Humidity: 62% to 71.5%	➢ Der p1^a^ ➢ Der f 1^a^ ➢ *E. maynei* ➢ *B. tropicalis* ➢ *B. kulagini* ➢ *B. tjibodas* ➢ *L. destructor* ➢ *G. privatus* ➢ *C. arcuatus* ➢ *T. putrescentiae* ➢ *S. medanensis*	• ELISA • Microscopy	(40)
Central America	Puerto Rico, USA	Houses (mattress and bedside floor)	Mean Temperature: 28 °C Mean Humidity: 75%	➢ Der p1^a^ ➢ Der f 1^a^	• ELISA	(41)
South America	Brazil	Houses (mattress)	Temperature: 25.1 °C to 25.5 °C Humidity: 78.5% to 82.5%	➢ Blo t 5^a^ ➢ Der p 1 ➢ *T. putrescentiae* ➢ *D. farinae* ➢ *A. siro*	• ELISA • Microscopy	(42)
		Houses of allergic patients (floor, sofa, and rug)	Temperature: Not mentioned Humidity: Not mentioned	➢ *D. pteronyssinus* ^a^ ➢ *E. maynei* ➢ *T. putrescentiae* ➢ *B. tropicalis* ➢ *Glycyphagus destructor* ➢ *G. domesticus* ➢ *Chortoglyphus arcuatus*	• Microscopy	(43)
		Hotels (mattresses, pillows and carpets)	Temperature: Not mentioned Humidity: Not mentioned	➢ Der f 1^a^ ➢ Der p1	• ELISA	(44)
		Houses (Bedroom and living room window curtains)	Temperature: Not mentioned Humidity: Not mentioned	➢ *D. pteronyssinus*^a^ ➢ *D. farinae* ➢ *E. maynei* ➢ *P. africanus* ➢ *B. tropicalis* ➢ *T. putrescentiae*	• Microscopy	(45)
		Car (seats)	Temperature: Not mentioned Humidity: Not mentioned	➢ Der 1	• ELISA	(46)
Southern North America	Mexico	264 Homes (bedroom floor and bed)	Temperature: Not mentioned Humidity: Not mentioned	➢ Der p 1 ➢ Der p 2 ➢ Der f 1	• Fluorescent multiplex bead-based assay	(47)
West Africa	Africa	Food stored grain	Temperature: 24.0 °C to 32.0 °C Humidity: 60% to 95%	➢ *Acarus farris* ➢ *Acarus siro* ➢ *Acarus sp.* ➢ *Rhyzoglyphus echinopus* ➢ *R. minutus* ➢ *Tyrophagus longior* ➢ *T. putrescentiae* ➢ *Glycyphagus destructor* ➢ *G. domesticus* ➢ *D. farinae* ➢ *Suidasia nesbitti* ➢ *Suidasia sp.*	• Microscopy	(48)

a Indicates the dominant dust mite species and allergen content based on reported abundance or prevalence in the cited study.

In Southeast Asia, countries such as Malaysia, the Philippines and Singapore consistently reported *B. tropicalis* as a common and abundant species in human dwellings, particularly in mattresses, sofas, carpets and bedroom floors, accounting for approximately 53% to 87% of the total mites (11, 15, 32). In contrast, studies conducted in hospitals in Singapore and Thailand reported higher concentrations of the major *Dermatophagoides* allergens, Der p 1 and Der f 1, in mattresses, carpets, and curtains, respectively (33, 36). Although these findings indicate increased patient exposure to *Dermatophagoides*-derived allergens in hospital settings, the use of ELISA that target only *Dermatophagoides*-specific allergens limits the detection of other common mite species, such as *B. tropicalis.*

Comparative studies between tropical (Indonesia, Malaysia, Thailand, and Vietnam) and subtropical regions (South Korea) have examined detection rates of major *Dermatophagoides* allergens in bedding dust, revealing higher levels in tropical countries (49). Higher HDM allergen levels were significantly observed in Southeast Asian countries, possibly reflecting the consistently warm, humid climate, which provides optimal conditions for mite survival and reproduction. This may contribute to higher allergen exposure and a greater prevalence of HDM-related allergic diseases compared with the subtropical region, though further clinical validation is required.

Large countries such as India and China encompass both tropical and subtropical zones, resulting in region-specific mite compositions. In tropical areas such as South Assam, India and Xishuangbanna Dai, China, dust sample were collected from houses of suspected patients with case history of allergic disease (26, 27). *Dermatophagoides* species are predominantly found in the sample, although *B. tropicalis*, *Acarus* spp., *Tyrophagus putrescentiae,* and *Aleuroglyphus ovatus* have also been identified. Similarly, studies from South America, such as Brazil and Ecuador, reported Der p 1, Der f 1 and Blo t 5 as predominant mite allergens collected in dust samples of individual mattresses (40–44). Other reported species include *Euroglyphus maynei, Blomia* spp*., Lepidoglyphus destructor, Glycyphagus* spp*., T. putrescentiae, Acarus siro, and Suidasia pontifica,* identified primarily using microscopy.

In Mexico, the study found that indoor allergen exposure was highly prevalent, with 85% of households having detectable levels of at least one allergen and over half exhibiting exposure to multiple allergens (47). Dust mite allergens (Der p 1, Der p 2) were among the most frequently detected, and allergen levels were significantly influenced by household and sociodemographic factors, including home size, pesticide use, cleaning practices, and occupancy characteristics. Similarly, environmental conditions such as indoor temperature and humidity also play a crucial role in shaping mite proliferation and allergen exposure (43). For example, a study in southern Brazil demonstrated that mite species composition and abundance varied among households from different socioeconomic groups, with higher mite diversity and density observed in the low-income group, which was characterized by favourable temperature and humidity. Interestingly, *D. farinae* was not detected in any of the samples, suggesting possible geographical variation in mite species distribution. In addition to household environments, exposure in public settings may also contribute to allergen burden; for example, a study in Brazil reported that taxis, but not private cars, acted as reservoirs of mite allergens, with group 1 *Dermatophagoides* allergens (Der 1) detected in 42% of taxi samples compared with only 5% in private cars, highlighting the role of frequently used and poorly ventilated environments in allergen accumulation (46). In Bloemfontein, South Africa, house dust mites were detected in 30% of homes sampled over one year, with *D. farinae* constituting approximately 97.5% of the mites found, despite outdoor climatic conditions being unfavourable (winter) for mite proliferation (50). The presence of HDMs was attributed to indoor microclimates created by factors such as artificial heating and poor ventilation during the winter months, suggesting that indoor environmental conditions can enable mite persistence even in dry climates.

Studies across tropical and subtropical regions show that house dust mite distribution is strongly shaped by indoor conditions such as temperature, humidity, and ventilation. Mites can persist even in unfavourable outdoor climates, leading to widespread allergen exposure in homes and public spaces. Public health strategies should focus on improving indoor environments, maintaining cleanliness, and raising awareness of mite reservoirs to reduce exposure. To mitigate the risk of mite-related allergic diseases, public health strategies should focus on controlling indoor humidity, improving ventilation, maintaining clean living environments, and raising awareness about mite reservoirs in both homes and public spaces, such as taxis.

Cross-habitat presence between HDMs and SMs is well documented. For example, HDMs *D. farinae* have been detected in stored food products and implicated in oral mite anaphylaxis (OMA), while SMs such as *T. putrescentiae* and *A. siro* are often found in domestic environments, contributing to respiratory allergies (51, 52). In Malaysia, SMs such as *S. pontifica*, *Austroglycyphagus sp*., *A. ovatus*, and *T. putrescentiae* have been reported in various food and industrial products, although no local cases of OMA have yet been documented (12, 25, 29). Notably, *D. farinae* contamination in wheat flour has been linked to anaphylaxis cases in Thailand and Singapore following ingestion of mite-contaminated food (34, 37). These findings highlight the ecological overlap between HDMs and SMs and emphasise the need for broader allergen surveillance and clinical awareness, particularly regarding food-related mite exposure.

Studies investigating the ecology and distribution of dust mites in tropical regions have employed different methodological approaches depending on their specific objectives. Conventional methods for determining the distribution of dust mites in specific ecological settings rely on microscopy and ELISA-based immunoassays. Microscopy remains useful for morphological species identification (53), but it is labour-intensive and provides no information on allergen content, which requires immunological or proteomic analyses (54). Conversely, immunological assays such as ELISA offer high sensitivity and specificity for quantifying major allergens such as Der p 1, Der f 1, and Blo t 5, but are limited to known targets and cannot detect novel or uncharacterised allergens (30–41). Therefore, combining both morphological and immunological approaches together with molecular tools such as DNA barcoding or multiplex qPCR is essential for a more accurate assessment of dust mite exposure and allergen diversity (55). Such integrated approaches are particularly important in tropical regions, where high species diversity and environmental overlap between HDMs and SMs complicate accurate allergen surveillance and diagnosis.

## 4 Prevalence of dust mite allergens in tropical regions

### 4.1 Southeast Asia, India and China

The prevalence of dust mite sensitisation in tropical regions is notably high, as persistently elevated humidity supports a larger population of mites and therefore higher concentrations of mite allergens in household dust (56). More than half of patients with allergic rhinitis and asthma in the tropics exhibit sensitisation to HDMs, particularly *D. pteronyssinus, D. farinae*, and *B. tropicalis* (Table 2). *B. tropicalis* is notably dominant in Southeast Asia, where it frequently serves as a major sensitising allergen, sometimes surpassing that of *Dermatophagoides* allergens (57–59).

**Table 2:** The prevalence of dust mite sensitisation in the tropical region.

Country	Study population and sample size	Diagnosed symptom	Diagnostic method	Allergen extracts/Components used	Sensitisation, n (%)	References
Indonesia	88 adult patients in Cipto Mangunkusumo Hospital, Jakarta.	Allergic asthma with/without allergic rhinitis.	*In vitro* - Specific specific IgE test	Purified recombinant allergens	*D. farinae –* 54 (62.1) *D. pteronyssinus* – 45(51.7) *Blomia tropicalis*-42 (48.3)	(60)
	380 students of secondary school in Depok and Bandar Lampung.	Allergic asthma or allergic rhinitis.	*In vivo* - Skin prick test	Purified allergen extracts (ALK-Abello, Hørsholm, Denmark)	Der f 1 - 90 (23.7) Der p 1 - 82 (21.6)	(61)
Philippines	258 children and adults from the University of Santo Tomas Hospital (USTH)	All forms of allergies.	*In vitro*- ELISA	*Suidasia pontifica* (*Sp*) extract	*Sp* extract – 100 (50)	(62)
	171 children and adults from Ilocos Region.	Asthma, allergic rhinitis, and/or atopic dermatitis.	*In vitro*- ELISA	• *Blomia tropicalis* (Bt) extract • Recombinant blo t 5 (rBlo t 5)	Bt extract- 99 (58) rBlo t 5–137 (80)	(63)
Brunei	223 patients	All forms of allergies.	*In vitro* (fluoroenzyme immunoassay (Phadia 100 platform)	Purified allergen extracts	Der p 1–100 (44.84) Der f 2–89 (39.91) Blo t 5- 65 (29.15)	(64)
Myanmar	111 patients in Rangoon (Yangon)	Asthma	*In vivo* - Skin prick test	Antigen (Der p 1) Bencard (Brentford, England).	Der p 1–55 (49.55)	(65)
Singapore	391 individuals	Atopy (atopic eczema, allergic rhinitis, and bronchial asthma)	• *In vivo* – skin prick test • *In vitro*- Fluorescence allergosorbent test (FAST)	Commercial extract (Greer Laboratories, USA) • *D. pteronyssinus* • *D. farinae* In-house allergen extract • *A. malaysiensis* • *B. tropicalis* • *S. brasiliensis* • *T. putrescentiae*	Skin prick test • *D. pteronyssinus-* 303 (77.49) • *D. farinae –* 300 (76.73) • *A. malaysiensis –* 253 (64.71) • *B. tropicalis –* 317 (81.07) • *S. brasiliensis –* 237 (60.61) • *T. putrescentiae –* 239 (61.13) FAST (Serum samples of 216 atopic) • *D. pteronyssinus -* 171 (79.2) • *D. farinae –* 162 (75.0) • *A.malaysiensis –* 148 (68.5) • *B. tropicalis –* 177 (81.9) • *S. brasiliensis –* 139 (64.4) • *T. putrescentiae –* 125 (57.9)	(15)
	60 patients	Asthma and Eczema	• *In vivo* – Skin prick test (SPT) • *In vitro* - ELISA	SPT-Commercial extract (Greer Laboratories, USA) • *D.pteronyssinus* • *D. farinae* ELISA Recombinant • Der p1 • Der p2 • Derp5 • Blo t4 • Blo t5 • Blo t6 • Blo t12 • Native Blo t 11	Skin prick test • *D.pteronyssinus* 30 (100) • *D. farinae* 30 (100) ELISA • Der p1 - 23 (76.7) • Der p2 – 22 (73.3) • Derp5 - 20 (66.7) • Blo t4 – 19 (63.3) • Blo t5 – 29 (96.7) • Blo t6 – 19 (63.3) • Blo t12- 21 (70.0) • Native Blo t 11–26 (86.7)	(58)
	206 Chinese individuals born in Singapore		*In vitro* - specific IgE (sIgE) ImmunoCAP (Phadia, Sweden)	• *D. pteronyssinus* • *B. tropicalis*	• *D. pteronyssinus –* 141 (68.5) • *B. tropicalis –* 142 (68.9)	(57)
Malaysia	85 patients	Allergic rhinitis	*In vivo* – Skin prick test (SPT)	*S. pontifica* extract	*S. pontifica* – 63 (74.1)	(66)
	100 adult patients in Loh Guan Lye Specialist Centre, Penang	Allergic asthma or allergic rhinitis.	*In vitro* - ELISA	In-house extract • *D. pteronyssinus* • *D. farinae* • *B. tropicalis* • *G. malaysiensis* • *A. ovatus* • *T.putrescentiae*	• *D. pteronyssinus –* 6(6) • *D. farinae –* 35 (35) • *B. tropicalis* – 5 (5) • *G. malaysiensis –* 37 (37) • *A. ovatus –* 4 (4) • *T.putrescentiae –* 2 (2)	(67)
	695 office workers	Allergic asthma or allergic rhinitis.	*In vivo* – Skin prick test (SPT)	Skin prick test kits (ALK Abello SA, Madrid, Spain) for	• *D. pteronyssinus –* 233 (33.53) • *D. farinae –* 227 (32.66)	(31)
Thailand	100 patients	Allergic Rhinitis	*In vivo* – Skin prick test (SPT)	SPT-Commercial extract (Greer Laboratories, USA)	• *D. pteronyssinus* – 76 (76) • *D. farinae* – 79 (79)	(68)
	1,393 Pediatric patients (age ≤15 years)	Asthma and/or allergic rhinitis	*In vivo* – Skin prick test (SPT)	SPT-Commercial extract	• *D. pteronyssinus* – 696 (49.96) • *D. farinae* – 669 (48.03)	(69)
Vietnam	610 patients at Pham Ngoc Thach (PNT) hospital, Southern Vietnam	Chronic respiratory diseases	*In vivo* – Skin prick test (SPT) *In vitro* - specific IgE	• Commercial airborne allergens (Stallergenes, France) • ImmunoCAP	Skin prick test • *Blomia tropicalis-* 114 (18.7) • *Dermatophagoides pteronissinus -* 109 (17.9) Specific IgE • *Blomia tropicalis-* 207(34) • *Dermatophagoides pteronissinus* – 250 (41)	(70)
	423 patients at the Unit of Allergy and Clinical Immunology, University Medical Center	Urticaria, Allergic Rhinitis and Atopic Dermatitis	*In vivo* – Skin prick test (SPT)	Standardised allergen (Starllergenes Greer, United Kingdom)	• *Dermatophagoides farinae* - 253 (59.8) • *Dermatophagoides pteronyssinus* - 213 (50.40) • *Blomia tropicalis* - 210 (49.6) • Storage mite mixed - 44 (10.40)	(71)
India	372 patients at the Allergy and Asthma Research Center, Kolkata.	Allergic rhinitis, bronchial asthma, atopic dermatitis and conjunctivitis.	*In vivo* – Skin prick test (SPT)	Commercial allergens (Merck, Germany)	• *Dermatophagoides pteronyssinus* – 302 (81.21) • *D. farinae* 327(87.87) • *Blomia tropicalis -* 276 (74.24) • *Acarus siro –* 123 (33.03) • *Lepidoglyphus destructora –* 94 (25.15) • *Tyrophagus putrescentiae –* 70 (18.78)	(72)
	605 patients at Allergy and Asthma Research Center, West Bengal.	allergic rhinitis, bronchial asthma and atopic dermatitis.	*In vivo* – Skin prick test (SPT)	Aeroallergen extracts (Credisol®, Mumbai, India)	• *Dermatophagoides pteronyssinus* – 486 (80.34) • *D. farinae –* 514 (84.92)	(73)
Sri Lanka	156 patients at the asthma clinic at the Professorial Unit Lady Ridgeway Hospital	Allergic rhinitis and rhinoconjunctivitis	*In vivo* – Skin prick test (SPT)	Aeroallergens (Allergropharma, UK)	• *Dermatophagoides pteronyssinus-* 59 (37.8)	(74)
China	45 patients with doctor-diagnosed asthma and rhinitis at Chengdu	Asthma Asthma and rhinitis	*In-vivo*- Skin prick test (SPT)	Recombinant dust mite allergens Blo t 4 and Blo t 5	• Blo t 4- 28 (62) • Blo t 5- 22 (49)	(75)
	244 subjects with suspected allergy symptoms from Northern, Central, and Southern China	Allergic rhinitis Allergic rhinitis and asthma Atopic dermatitis	*In vitro-* Protein chip technology	Der p 1, Der f 2, Der p 2, Der f 1, Der p 23, Der p 21, Der p 7, Der p 5, and Der p 10	• Der p 1- 161 (66) • Der f 2- 145 (59.4) • Der p 2- 144 (59) • Der f 1- 119 (48.8) • Der p 23- 111 (45.5) • Der p 21- 57 (23.3) • Der p 7- 55 (22.5) • Der p 5- 37 (15.2) • Der p 10- 22 (9)	(76)
Africa	1 671 patients	Asthma or clinical/treated asthma or wheezing/whistling breath	*In vivo* – Skin prick test (SPT)	Allergens (Immunospec [Pty] Ltd, Johannesburg, Gauteng, South Africa)	• House dust mite mix *–* 1103 (66) • *B. tropicalis –* 1036 (62)	(22)
South Africa	166 black South African children	Atopic dermatitis	*In vitro-* Customised allergen chip (ISAC technology)	Der p 1, Der p 2, Der p 23	• Der p 1 – Urban 93 (56), Rural 75 (45) • Der p 2 – Urban 110 (66), Rural 105 (63) • Der p 23 – Urban 83 (50), Rural 81 (49)	(77)
Cameroon	201 asthmatic adolescents and adults	Asthma	*In vivo* – Skin prick test (SPT)	Standardised allergenic extracts of Stallergenes Laboratories (Anthony, France)	• *Dermatophagoides pteronyssinus – 107* (53.2) • *Dermatophagoides farinae -* 100 (49.8) • *Blomia tropicalis* - 96 (47.8)	(78)
Jamaica	160 individuals	Asthma, atopy and sickle cell disease (SCD)	*In vivo* – Skin prick test (SPT)	Standardized commercial extracts (HollisterStier Laboratories, Spokane, WA, USA 99220)	• *Dermatophagoides* *Pteronyssinus -* 53 (33) • *Dermatophagoides farinae -* 52(32*)*	(79)
Nigeria	346 children	-	*In vivo –* Skin prick test (SPT)	Extracts from ALK-Abello, Horsholm, Denmark.	• *D. pteronyssinus* – 30 (8.6) • *D.farinae* – 25 (7.2)	(80)
South Africa	587 schoolchildren	Respiratory, rhinoconjunctival and cutaneous symptoms	*In vivo –* Skin prick test (SPT)	Extracts from ALK-Abello, Horsholm, Denmark.	• *Der* (Mix of *D. pteronyssinus* and *D. farinae*) – 340 (58) • *B. tropicalis –* 58 (9.9)	(81)
Cuba	148 patients	Asthma	*In vivo* – Skin prick test (SPT)	In-house extract *D. siboney* and *B. tropicalis* Commercial extract (Soluprick@ SQ, Denmark). D. *pteronyssinus, D. farinae, A. siro,* and *L. destructor*	• *D. siboney -* 130 (88) • *D. pteronyssinus -* 129 (87) • *A. siro -* 126(85) • *B. tropicalis -* 126(85) • *D. farinae -* 123(83)	(93)
Barbados	481 patients	Asthma	*In vitro* - specific IgE	Immunochemiluminometric Magic Lite assay (Magic Lite Total IgE Extended Range; Ciba-Corning, Medfield, Mass., USA)	• Der p 1–265 (55) • Blo t 5–313 (65)	(17)
Costa Rica	283 children at Hospital Nacional de Ni∼nos	history for asthma treatments, family history for allergic disorders, and environmental tobacco smoke (ETS) exposure at home.	*In vitro* - specific IgE	Phadia ImmunoCAP assay (Phadia, Uppsala, Sweden).	• *D. pteronyssinus –* 208(73.5) • *Dermatophagoides* *farinae –* 210 (74.2) • *Blomia tropicalis –* 211(74.6)	(82)
Brazil	74 patients	Group I - patients with atopic dermatitis and other allergic respiratory diseases, such as allergic rhinitis and/or asthma. Group II - patients with only respiratory diseases, such as rhinitis and/or asthma, without atopic dermatitis.	*In vivo* – Skin prick test (SPT)	Extracts from International Pharmaceutical Immunology ASAAC Brasil (IPI ASAAC)	• *Dermatophagoides pteronyssinus* 66 (89.2) • *Dermatophagoides farinae* 55 (74.3) • *Blomia tropicalis -* 21 (28.4)	(83)
	108 patients at Hospital da Crianc	Allergic diseases	*In vivo* – Skin prick test (SPT)	Allergen extract (Immunotech-FDA Allergenic LTDA)	• *Dermatophagoides pteronyssinus* - 45 (42) • *Dermatophagoides farinae* - 40 (37) • *Blomia tropicalis -* 36 (33)	(84)
Venezuela	115 subjects who attended the Allergy Clinic, Institute of Biomedicine, Caracas.	Asthma and/or allergic rhinitis	• *In vivo* – Skin prick test (SPT) • *In vitro* - ELISA	In-House allergen extract	Skin prick test • *B. tropicalis* 94 (81.74) • *D. pteronyssinus* 93 (80.87) ELISA • *B. tropicalis* 94 (81.74) • *D. pteronyssinus* 93 (80.87)	(85)
	229 patients	Rhinitis or rhinosinusitis	*In vivo* – Skin prick test (SPT)	Mite extracts were provided by Laboratorios Diater (Buenos Aires, Argentina)	• *D. farinae –* 208 (91) • *D. pteronyssinus –* 206 (90) • *B. tropicalis -* 169 (74) • *G. domesticus* - 140 (61) • *C. arcuatus -*135 (59) • *A. siro -*108 (47) • *L. destructor* - 82 (36) • *T. putrescentiae* - 82 (36)	(86)
Peru	268 subjects	Allergic rhinitis (AR) and/or asthma	• *In vivo* – Skin prick test (SPT) • *In vitro* - ELISA	• Standardized allergen extracts (Inmunotek Madrid, Spain). • ALEX MacroArray diagnostic system (MacroArray Diagnostics, Vienna, Austria).	Skin prick test *Dermatophagoides pteronyssinus, D. farinae Lepidoglyphus destructor, Blomia tropicalis -* 229 (85.45) ELISA Der p 1, Der p 2, Der p 5, Der p 7, Der p 10, Der p 11, Der p 20, Der p 21, Der p 23, Der f 1, Der f 2, Blo t 5, Blo t 10, Blo t 21, Lep d 2, Gly d 2, Tyr p 2 - 158 (58.95)	(87)
Colombia	97 asthmatic patients	Asthma	*In vitro* - Radioallergosorbent (RAST)	In-House allergen extract	• *Suidasia medanensis –* 71 (73.2%)	(88)
	61 patients	Severe asthma	*In vivo* – Skin prick test	Standardised allergen extracts	• *Dermatophagoides pteronyssinus -* 35 (56.9*)* • *Dermatophagoides farinae -* 43 (70.7) • *Blomia tropicalis -* 12 (19) • *Lepidoglyphus destructor -* 15 (24.2)	(89)
	127 children at Hospital Infantil Napoleon Franco Pareja	wheezing	• *In vivo* – Skin prick test (SPT) • *In vitro* - ImmunoCAP	• Inmunotek (Madrid,Spain) • ImmunoCAP (Thermo Fisher, Uppsala, Sweden)	Skin prick test • *Dermatophagoides pteronyssinus, D. farinae* and *Blomia tropicalis -* 8 (9.7) ImmunoCAP • *Blomia tropicalis and Dermatophagoides pteronyssinus -* 23 (31%)	(90)
	66 adults in Bogota	Severe asthma	• *In vivo* – Skin prick test (SPT)	Standardised allergen extracts	• *Dermatophagoides pteronnysinus -* 53 (80.3) • *Dermatophagoides farinae -* 52 (78.8) • *Blomia tropicalis -* 45 (68.2)	(91)
Spain and Latin America (Colombia, Costa Rica, and Guatemala)	218 allergic patients in Spain (n = 130) and in 3 countries in Latin America (n = 88)	Rhinitis/rhinoconjunctivitis and/or asthma	*In vitro* - specific IgE	ImmunoCAP, (Thermo Fisher Scientific).	• *D pteronyssinus –* 196 (90) • *Dermatophagoides farinae* 174 (80) • Der p 1*-* 159 (73) • Der p 2–172 (79) • Der p 23–150 (69*)*	(92)

In Singapore, studies have included other allergens besides *Dermatophagoides* allergens (Der p 1 and Der f 1) when investigating HDM sensitisation patterns. For example, one study compared sensitisation profiles among children with asthma and eczema to *D. pteronyssinus* and *B. tropicalis* and their specific allergens (58). The asthma group showed the highest sensitisation to Blo t 5 (96.7%), while the eczema group exhibited greater reactivity to Der p 5. Another study shows similar findings: children with atopic dermatitis show a preferential sensitisation to *Dermatophagoides* mites, which is negatively associated with *B. tropicalis* (93). Both studies showed that asthma and eczema patients exhibit distinct HDM sensitisation profiles, suggesting differing underlying allergic mechanisms. Additionally, studies have also demonstrated that over 80% individuals living in Singapore displayed monospecific IgE sensitisation to Der p and Blo t, with HDM-specific IgE titres far exceeding those for other allergens (57). These findings indicate that frequent exposure to certain HDMs in the urban tropical environment may play a significant role in shaping sensitisation patterns and allergic airway disease in Singapore. A comparative study between Singapore (*n* = 203) and Taiwan (*n* = 60) further reported similar skin test reactivity frequencies to *D. pteronyssinus* (97.5% vs. 88.3%), but higher reactivity to *B. tropicalis* in Singapore (93.1% vs. 73.3%), likely reflecting greater environmental exposure among Singaporeans (16). Although the overall frequency of mite sensitisation is comparable in Singaporean and Taiwanese allergic populations, the pattern and intensity of sensitisation differ. Furthermore, the same study also reported that the domestic mite densities in Singapore were 76-fold higher than the numbers found in Taiwan. These differences likely reflect regional differences in mite abundance, with sustained high mite levels in Singapore contributing to increased allergen exposure.

In Malaysia, although *B. tropicalis* is abundant in dust samples (8,934 mites per gram of dust), epidemiological data on its clinical sensitisation remain limited (11, 24). Reports from a specialist centre in Penang indicated a low sensitisation rate (5%) to *B. tropicalis* extract, while another study at the University of Malaya Hospital found a much higher prevalence (56%) to Blo t 5 (59, 67). The differences in sensitisation rates may be due to variations in study populations or the types of allergen extracts used. These findings highlight the need for standardised testing methods and multicentre studies to obtain more consistent and comparable data. A comparative study was conducted to identify common aeroallergens between two countries with contrasting economies, lifestyles, and climates: Malaysia and the Netherlands (94). HDM were identified as the most common aeroallergen in both regions. Notably, Malaysian participants showed significantly higher rates of aeroallergen sensitisation compared to the Netherlands, particularly to HDMs (82.4% vs. 41.2%), with 69%–78% reacting to *D. pteronyssinus*, *D. farinae*, and *Blomia* species. The higher sensitisation in Asia is expected due to its humid and moderate climate. In temperate regions, HDM populations fluctuate seasonally, though indoor microclimates allow limited survival during colder months (95). Although the study does not compare genetic factors between populations in Malaysia and the Netherlands, host genetic variability may also contribute to differences in allergen sensitisation. Variations in immune response genes, including differences in the composition of HLA alleles, may influence individual responses to HDM allergens (96, 97). Therefore, future prospective studies are needed to further investigate the role of genetic factors that may contribute to the differences in sensitisation patterns.

Beyond Southeast Asia, similar patterns of dust mite sensitisation are observed in South Asia countries such as India and Sri Lanka, where different regions show variable dominance between *D. pteronyssinus* and *D. farinae*, along with significant sensitisation to *B. tropicalis* and other domestic mites (72–74). A study in Kolkata, India, evaluated sensitisation to HDMs and SMs among 372 patients with allergic rhinitis, asthma, atopic dermatitis, or conjunctivitis to address the limited data on SM sensitisation (72). High sensitisation rates were observed for *D. farinae* (87.9%), *D. pteronyssinus* (81.2%), and *B. tropicalis* (74.2%), while substantial sensitisation was also detected for SMs, including *A. siro* (33%), *L. destructor* (25%), and *T. putrescentiae* (18%). Kolkata has a tropical monsoon climate characterised by high humidity and intense rainfall. The high sensitisation rates to both HDMs and SMs may be attributed to elevated indoor humidity and the presence of grain storage within patients' homes. The findings support the inclusion of SMs in routine skin prick testing in this region, where high indoor humidity and household grain storage may contribute to increased SM exposure. Similarly, a retrospective analysis of an atopic population of West Bengal was conducted to investigate the sensitisation to common aeroallergens such as pollens, molds, and HDMs (73). The results show that *D. pteronyssinus* and *D. farinae* had the highest sensitisation rates compared to other allergens at 80.34% and 84.92%, respectively.

A multi-centre study in China demonstrated that molecular sensitisation to house dust mite (HDM) components varies across populations, with key allergens such as Der p 1, Der p 2, and Der p 23 frequently identified as major IgE-binding components (76). The study highlights that component-resolved profiles provide a more detailed understanding of sensitisation patterns beyond extract-based testing, particularly in a geographically diverse country like China, where allergen exposure differs by region. This geographical influence is further supported by a study on *B. tropicalis* allergen Blo t 4, which showed marked regional differences in allergenicity (75). Sensitisation to Blo t 4 was significantly higher in subjects from Chengdu, China (28%), compared to those from Singapore (4%), despite both being in Asia. Interestingly, Blo t 4 showed higher sensitisation than the major allergen Blo t 5 in the Chinese cohort, suggesting that certain allergen components may be more clinically relevant in specific regions. Together, these findings indicate that mite sensitisation profiles in China are strongly influenced by geographical and environmental factors, which may affect both the dominant allergen components and their clinical relevance. This supports the need for region-specific molecular diagnostic approaches to accurately characterise sensitisation patterns and guide appropriate management strategies.

### 4.2 Regional sensitisation in Latin America and Africa

In Latin America, a study in Brazil highlights the complexity of HDM sensitisation in tropical regions, where both immediate and delayed hypersensitivity responses coexist using three common house dust mites (*D. pteronyssinus*, *D. farinae*, and *B. tropicalis*) in 74 patients with respiratory allergies, with or without atopic dermatitis (83). Importantly, the study revealed substantial overlap between immediate (skin prick test) and delayed (atopy patch test) hypersensitivity responses, with 71.6% of patients positive in both tests. However, a subset of patients (8%) showed positivity only in the atopy patch test (APT) despite negative skin prick results, indicating that APT can detect additional sensitisation not captured by conventional testing and highlights the diagnostic value of APT as a complementary tool, particularly in identifying delayed-type hypersensitivity to HDM allergens in patients with respiratory allergic diseases. Furthermore, the findings reported by Araujo et al. (2019) revealed that HDM sensitisation among children and adolescents (Derp-42%, Derf-37% and Blot-33%) in the northeast of Brazil is not solely driven by climatic conditions but is strongly modulated by socio-environmental factors (84). While high humidity and temperature in northeastern Brazil create favourable conditions for mite proliferation, the persistence of sensitisation is closely linked to housing quality, ventilation, and socio-economic status. This suggests that allergen exposure in such settings is amplified by indoor environmental conditions rather than climate alone. Importantly, these findings challenge the assumption that tropical sensitisation patterns are uniform, emphasizing instead that intra-regional variability is shaped by differences in living conditions. Consequently, effective management of HDM-related allergies in tropical regions requires not only clinical intervention but also improvements in housing and environmental control strategies.

Across Africa, a consistently high prevalence of atopy is reported, with house dust mites emerging as dominant sensitizing agents and key contributors to asthma morbidity. In one cohort of 160 individuals recruited irrespective of asthma history, sensitisation rates of 32% to *D. farinae* and 33% to *D. pteronyssinus* were observed, highlighting the widespread burden of HDM sensitisation in the general population (79). High IgE sensitisation rates were also observed among urban and rural children in South Africa, with HDMs as the dominant allergens (81% urban, 74% rural) (77). The major components, such as Der p 1 and Der p 2, showed the highest reactivity in both settings, while Der p 23 further contributed to the sensitisation profile. Notably, even very young children had already developed strong IgE sensitisation to HDM, indicating early-life exposure and rapid immune priming. Furthermore, among asthmatic populations in East Africa, polysensitisation to major allergens, including house dust mites (66%), *Blomia tropicalis* (62%), and cockroach (52%), was associated with reduced lung function and increased healthcare utilization, although its relationship with asthma control and severity remains complex (22).

In Sub-Saharan Africa, the patterns of house dust mite (HDM) sensitization are intricately linked to socioeconomic transitions and environmental exposures. Research in north-central Nigeria demonstrated that urbanization and higher family affluence are significant drivers of atopy, with urban children showing a sensitization rate of 15.6% compared to just 2.8% in rural areas (80). This trend is further complicated by the high prevalence of helminth infections in tropical regions, which may exert an immunomodulatory effect on allergic responses. For instance, a study in Gqeberha, South Africa, found that while HDM remained the most common allergen (sensitization rate of 14.8%), there was a complex relationship between parasite infections and atopy (81). Interestingly, children infected with *Ascaris lumbricoides* showed a significant association with increased HDM-specific IgE levels, suggesting that in some African contexts, certain parasitic infections may actually enhance, rather than suppress, the allergic inflammatory response to mite allergens. These findings underscore the unique immunological landscape of the African tropics, where the “Hygiene Hypothesis” must be interpreted alongside high pathogen burdens. Collectively, these findings indicate that HDM sensitisation in Africa is shaped by a multifactorial interplay of early-life exposure, environmental conditions, and lifestyle factors, rather than climate alone.

A cross-regional synthesis of these data reveals that while high humidity is a universal driver of mite abundance, the specific dominance of *B. tropicalis,* particularly in Southeast Asia, compared to the emerging patterns in Africa and Latin America, suggests that tropical sensitization is not a monolithic phenomenon but is finely tuned by local microclimates and urbanization levels. Furthermore, the significant cross-reactivity between the *Dermatophagoides* and *Blomia* groups, combined with regional variations in molecular components such as Blo t 4 and Blo t 5, underscores a critical diagnostic gap: relying on temperate-climate allergen panels likely results in a substantial underestimation of the true allergic burden in tropical populations.

### 4.3 Storage mites in tropical regions

Storage mites (SMs) allergy often receives limited attention in allergology practice and is frequently overlooked in clinical diagnosis (98). Notably, many of the most clinically relevant storage mite species belong to the Acaridae family, commonly referred to as flour, grain, or cheese mites (10, 25, 99).

In a cohort of 200 consecutive patients from the Porto district with suspected allergic rhinitis and/or asthma who were evaluated in an outpatient clinic, 123 individuals (61.5%) showed positive sensitisation to at least one storage mite species (100). Among these, *Lepidoglyphus destructor* was the most prevalent (69.9%), followed by *T. putrescentiae* (50.4%), *B. tropicalis* and *G. domesticus* (48.8%), and *A. siro* (24.4%). These findings suggest that sensitisation to storage dust mites may be more important than previously recognised and should be considered in standard diagnostic assessment. Supporting this observation, an experimental study conducted in Korea compared airway inflammation induced by the storage mite *T. putrescentiae* with that caused by house dust mites (*D. farinae* and *D. pteronyssinus*) in a mouse asthma model (101). Although all mite species triggered airway inflammation, mice exposed to *T. putrescentiae* exhibited significantly greater airway resistance, higher numbers of inflammatory cells (including eosinophils and neutrophils), and more severe lung tissue damage. These mice also showed increased mucus hypersecretion and fibrosis, suggesting that storage mite sensitisation may induce stronger respiratory inflammatory responses than house dust mite exposure.

In addition to respiratory manifestations, SMs are implicated in food-related allergic reactions, most notably oral mite anaphylaxis (OMA), a severe IgE-mediated reaction triggered by ingestion of mite-contaminated flour or food products (52, 102, 103). Despite their clinical relevance, these findings underscore the importance of including storage mite allergens in diagnostic testing to ensure accurate identification of sensitisation and prevention of both respiratory and food-related allergic events.

Besides *T. putrescentiae* and *A. siro*, other SMs such as *Suidasia pontifica* (also referred to as *S. medanensis*), a SMs reported in several tropical countries and belong to the family Suidasiidae (previously Acaridae) (25, 77). This mite is commonly found in proximity to humans, such as in household environments and stored products, making it a significant allergen with notable economic and public health implications (11, 25). Remarkably, 74% of Malaysian (*n* = 85) and 73% of Colombian (*n* = 97) patients were sensitised to this mite, highlighting its emerging clinical importance (66, 88). These findings suggest that *S. pontifica* may represent an important but often overlooked allergen source in tropical regions, with implications for improving and developing diagnostic accuracy strategies. Recently, a study from the Philippines reported that *S. pontifica* is an allergenic mite species, with IgE-binding reactivity detected in 47% of sera from allergic patients and controls (*n* = 200), supporting its allergenicity and clinical relevance among atopic individuals (104). These findings further highlight the need to characterise *S. pontifica* allergens to elucidate their contribution to tropical allergen exposure.

### 4.4 Cross-sensitisation and urban–rural exposure

One of the key features of mite sensitisation in the tropics is that patients in tropical regions usually present sensitisation to more than one HDM allergen (31, 57). However, no studies currently demonstrate the exact overlap between HDMs and SMs. Most available studies focus on comparing HDM allergens with non-mite allergen sources, such as food, fungi, or pollen, rather than directly addressing the relationship between HDMs and SMs (84, 105). Therefore, future studies should investigate the prevalence of sensitisation to both house dust mites and storage mites among allergic patients to better understand their relative clinical relevance.

The pattern of mite exposure and sensitisation may differ between urban and rural settings due to variations in lifestyle and environmental conditions. For example, urban populations are generally more exposed to HDMs in bedding and furniture, whereas rural communities face additional exposure to SMs through agricultural and food-handling environments (31, 106). This dual exposure complicates diagnosis and reinforces the need for comprehensive allergen panels that include both HDM and SM components. These findings emphasise the need to broaden diagnostic and therapeutic approaches beyond *Dermatophagoides* models to ensure accurate assessment and effective management of allergic diseases in tropical populations. Taken together, regional variability in mite sensitisation across tropical regions underscores the need for standardised diagnostic strategies and inclusion of locally relevant mite species to better capture true allergenic exposure patterns.

## 5 Diagnostic paradigms in tropical house dust mite allergy

An accurate diagnosis is the cornerstone of effective allergy management, guiding both targeted allergen avoidance strategies and appropriate therapeutic interventions (21). The diagnostic process is a multi-step approach, classically built on three pillars: a detailed clinical history, a thorough physical examination, and targeted diagnostic testing (107). Although clinical history and physical examination are fundamental for identifying suspected allergic conditions, their diagnostic accuracy is limited because allergic symptoms often overlap with those of non-allergic disorders. Therefore, objective laboratory investigations are required to confirm allergen-specific IgE sensitisation and establish a definitive clinical correlation (108). The diagnostic tests for allergic diseases are broadly classified into *in vivo* tests, which measure a direct biological response within the patient, and *in vitro* tests, which analyse serum or blood samples for specific immunological markers (109).

Among *in vivo* methods, the skin prick test (SPT) is the most common and cost-effective tool for assessing IgE sensitisation in humans (22). It remains the preferred initial diagnostic method globally due to its high sensitivity, low cost, rapid results (typically within 15–20 min), and a minimally invasive procedure (54). The test is performed by introducing a minute quantity of standardised allergen extract into the epidermis. A positive result, observed as a wheal-and-flare response, provides a rapid, visible confirmation of IgE-mediated mast cell sensitisation (108).

**Figure 3:**
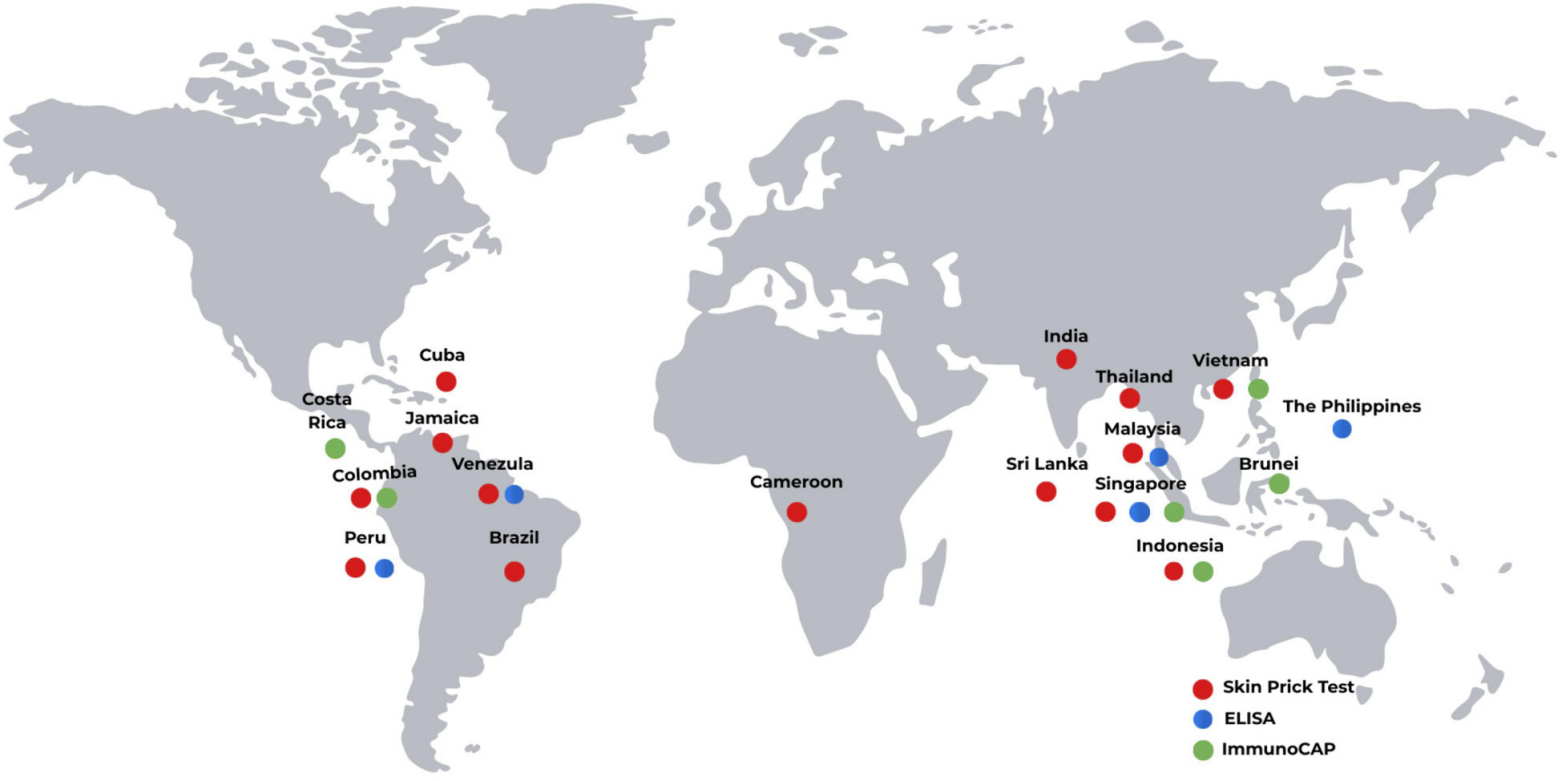
Geographic distribution of diagnostic methods for dust mite sensitisation in tropical countries. The map illustrates the primary diagnostic tools utilized in peer-reviewed literature to assess IgE-mediated sensitivity. **Red circles** indicate the use of *in vivo* Skin Prick Tests (SPT); **blue circles** represent *in vitro* Enzyme-Linked Immunosorbent Assays (ELISA) for total or specific IgE; and **green circles** denote the use of the ImmunoCAP automated system. The spatial distribution is based on the geographical locations of study populations reported in the literature included in this review (1990–2026).

Due to these advantages, SPT remains the primary diagnostic tool employed in most large-scale epidemiological studies assessing dust mite sensitisation prevalence across tropical regions (Figure 3). For example, extensive studies in Thailand and Vietnam have reported using SPT to demonstrate that sensitisation to *D. pteronyssinus, D. farinae* and *B. tropicalis* using standardised extracts (68–71). Similarly, countries such as India, Sri Lanka, Cameroon, Brazil, Jamaica, and Cuba have primarily reported the prevalence of dust mite sensitisation using SPT (72, 74, 78, 79, 83, 110). The continued reliance on SPT as the gold standard in these regions is mainly due to its practicality as a low-cost, rapid, and reliable screening tool suitable for large population studies. Consequently, there is relatively lack of reported research using alternative diagnostic approaches. These include conventional serum-specific IgE assays, such as ImmunoCAP or ELISA, and molecular/component-resolved diagnostics, such as microarrayed allergen profiling, multiplex immunoassays, or single recombinant allergen testing, which provide a more detailed understanding of sensitisation profiles (24).

Although SPT is a practical and effective screening method, it only reflects the biological response of IgE-mediated sensitisation and does not identify the specific allergen components involved (108). Moreover, SPT cannot be performed on patients with extensive skin conditions or those taking antihistamines (111). In such cases, *in vitro* diagnostic tests serve as suitable alternatives, allowing the quantification of serum-specific IgE and enabling the detailed identification of allergenic components (112). The most common *in vitro* approaches include enzyme-linked immunosorbent assay (ELISA) and commercially available immunoassay systems, such as ImmunoCAP, which measure specific IgE levels against selected allergen extracts or purified allergen molecules (23, 104).

In tropical countries, the *in vitro* assays have also been applied to evaluate mite sensitisation profiles at the molecular level. One study in Malaysia used ELISA and immunoblot techniques with crude mite extracts to assess IgE reactivity in allergic individuals (67). The result showed that adult allergic subjects had significantly higher mean serum-specific IgE levels to *D. farinae* (35%) and *Glycycometus malaysiensis* (37%). Immunoblot analysis indicated that not all allergic individuals displayed positive IgE reactivity to the tested mites extract. Sensitisation was most frequently associated with Group 2 (9–12 kDa), Group 10 (38 kDa), and Group 18 (40–48 kDa) allergens. Additionally, the study demonstrated distinct sensitisation profiles between HDMs and SMs, suggesting that most individuals were primarily sensitised to a single mite species. However, potential cross-reactivity between HDM and SM allergens may still occur due to the presence of conserved allergen families, such as tropomyosin (Group 10) and other homologous proteins, which share structural similarities across mite species. These findings highlight the complexity of mite sensitisation patterns and support the use of Component-Resolved Diagnostics (CRD) employing defined allergen components to better differentiate true sensitisation from cross-reactive responses.

Furthermore, in many studies, diagnostic analyses in tropical regions are restricted to a limited set of major mite allergens, mainly *D. pteronyssinus* (Der p 1 and Der p 2) and *Dermatophagoides farinae* (Der f 1 and Der f 2), while allergens from other clinically relevant tropical species, such as *B. tropicalis,* are less frequently included, typically represented only by Blo t 5 (7, 17). This limitation may lead to underestimation of sensitisation patterns and highlights the need for region-specific diagnostic panels. For example, a study involving 115 patients with a history of allergic respiratory disease in Venezuela investigated sensitisation profiles using both SPT and serum-specific IgE detection (85). In this study, SPT was performed using allergenic extracts of *B. tropicalis* and *D. pteronyssinus*, while serum IgE reactivity was evaluated using ELISA with mite allergen extracts. The results demonstrated that *B. tropicalis* was a major sensitising species (81.74%), with several patients exhibiting strong positive reactions in both SPT and ELISA. Notably, *B. tropicalis* had not been routinely included in earlier diagnostic test panels in Venezuela, which may have led to underestimation of its clinical relevance. Interestingly, a subset of individuals (*n* = 14) was found to be monosensitised to *B. tropicalis*, indicating that sensitisation was not solely attributable to cross-reactivity with *Dermatophagoides* species. These findings highlight the importance of incorporating *B. tropicalis* allergens into both *in vivo* and *in vitro* diagnostic panels to ensure accurate identification of sensitisation patterns and to improve diagnostic precision for allergic diseases in tropical regions.

Currently, 26 allergens have been identified for *B. tropicalis* according to the World Health Organisation/International Union of Immunological Societies (WHO/IUIS) Allergen Nomenclature Sub-Committee database. Several studies from the tropical region have reported that some *B. tropicalis* allergens play an important role in allergic reactions. A case-control study among Colombian populations demonstrated that IgE sensitisation to *B. tropicalis* allergens, particularly Blo t 5, Blo t 2, and Blo t 21, represents a key allergen component associated with asthma (113). Additional studies have further confirmed the clinical and immunological relevance of Blo t 5, Blo t 21, and Blo t 2 in respiratory allergic inflammation (7, 114).

Limitations associated with conventional diagnostic methods, including variability in allergen extract composition and inconsistent sensitivity of skin prick testing, have led to the increasing use of standardised *in vitro* platforms such as the ImmunoCAP system for the quantitative detection of allergen-specific IgE (108). This assay provides a highly standardised and reproducible method for evaluating sensitisation to specific allergens (115). In tropical regions where exposure to HDMs is high and multiple mite species coexist, ImmunoCAP can be widely applied to improve the detection and characterisation of mite sensitisation profiles. The ImmunoCAP system has been increasingly employed in several tropical countries as a standardised *in vitro* diagnostic platform to quantify allergen-specific IgE. For example, studies in Brunei assessed the sensitisation profiles of patients with allergic diseases using ImmunoCAP-based specific IgE detection, performed using a fluoroenzyme immunoassay (FEIA) on the Phadia 100 platform to examine both aeroallergens and food allergens (64). The study revealed that specific IgE (sIgE) levels to major dust mite species, including *D. pteronyssinus*, *D. farinae*, and *B. tropicalis*, were among the highest detected in the cohort. Additionally, the evaluation of HDM allergy in chronic respiratory disease patients in Vietnam found that sIgE was significantly more sensitive than SPT, detecting over twice as many cases of sensitisation (70). Nevertheless, because SPT results were still statistically associated with sIgE, SPT remains a recommended, viable screening method for chronic respiratory disease patients in Southern Vietnam.

Recent advances in molecular allergology have led to the introduction of CRD, an *in vitro* method that represents a major step forward in allergy diagnostics. This method utilises purified natural or recombinant allergen molecules instead of crude extracts to detect specific IgE (sIgE) antibodies in patient sera (116). This approach enables precise identification of sensitisation to individual allergen components, allowing clinicians and researchers to distinguish between genuine sensitisation to species-specific allergens and cross-reactivity arising from homologous proteins shared among different allergen sources (23). CRD can be performed through singleplex assays, such as ImmunoCAP or ELISA, which assess one allergen component at a time, or through multiplex platforms like ImmunoCAP ISAC and ALEX2, which analyse multiple allergenic molecules simultaneously (115). By providing a detailed molecular sensitisation profile, CRD offers higher diagnostic accuracy, enabling differentiation between primary sensitisation and cross-reactivity and supporting personalised allergen immunotherapy (AIT) across different geographic regions (117). Moreover, CRD provides significant advantages in guiding allergen immunotherapy by identifying clinically relevant allergen molecules responsible for patient sensitisation (116). Extract-based tests may detect IgE sensitisation but may not distinguish between true sensitisation and cross-reactivity, which can influence the accuracy of AIT selection. In contrast, CRD based on defined allergen molecules can help identify clinically relevant allergen components, thereby supporting a more targeted and personalised approach to AIT. For example, one study showed that patients who were sensitised to allergen molecules included in the AIT vaccine demonstrated better treatment responses compared with those sensitised to allergens that were absent from the vaccine formulation (118). These findings suggest that molecular profiling of allergen-specific antibody responses can help optimise AIT by ensuring that relevant allergen components are included in the therapeutic extract.

Despite its potential, CRD has several reported limitations. Its clinical application remains limited in some regions, such as China, due to the lack of available reagents for allergen components and the complexity of interpreting results among clinicians (119). In addition, commercially available CRD platforms cover only a limited number of allergen components and are associated with higher costs compared with conventional diagnostic methods (120). Although the skin prick test continues to serve as the primary diagnostic tool for allergy assessment in tropical regions because of its affordability and ease of use, it offers limited insight into the molecular basis of allergen sensitisation. *In vitro* techniques, including ELISA and ImmunoCAP, provide valuable complementary information by allowing quantitative measurement of allergen-specific IgE and revealing population-specific sensitisation profiles. More advanced molecular approaches, such as component-resolved diagnosis, enhance diagnostic accuracy by differentiating true sensitisation from cross-reactivity; however, their broader clinical adoption in tropical settings remains limited by high costs, limited availability of allergen components, and challenges in interpreting results.

## 6 Future research direction

House dust mite (HDM) allergy poses a significant public health issue in tropical regions, where constant warmth and humidity create ideal conditions for mite growth and ongoing allergen exposure. Current evidence from tropical studies highlights a diverse range of mite species. However, allergen characterisation is largely limited to *D. pteronyssinus*, *D. farinae*, and *B. tropicalis*, with scarce epidemiological and molecular data for other mite species. Variations in species distribution and allergen profiles across different countries emphasise the need for region-specific research to accurately characterise sensitisation patterns. These ecological and molecular insights are essential for understanding the clinical importance of local mite populations and their role in allergic conditions such as asthma, rhinitis, and dermatitis.

Despite advances in diagnostic technology, practices in many tropical areas often rely on traditional methods like the Skin Prick Test (SPT), which only indicate a biological response and lack molecular precision. Expanding the use of *in vitro* tests such as ELISA, ImmunoCAP, and component-resolved diagnosis (CRD) can enhance diagnostic accuracy by distinguishing genuine sensitisation from cross-reactivity and identifying clinically relevant allergen components.

Future research should prioritise the development of standardised ecological sampling and mite extracts, creating molecular allergen databases, and multicentre studies integrating ecological, molecular, and clinical data. This progress will allow for more accurate diagnosis, facilitate the development of regionally tailored immunotherapies, and ultimately reduce the burden of mite-related allergic diseases in tropical populations.
